# The DNA methyl-transferase protein DNMT1 enhances tumor-promoting properties of breast stromal fibroblasts

**DOI:** 10.18632/oncotarget.23411

**Published:** 2017-12-18

**Authors:** Layla A. Al-Kharashi, Falah H. Al-Mohanna, Asma Tulbah, Abdelilah Aboussekhra

**Affiliations:** ^1^ Department of Molecular Oncology, King Faisal Specialist Hospital and Research Center, Riyadh 11211, KSA; ^2^ Department of Pharmacology and Toxicology, Faculty of Pharmacy, King Saud University, Riyadh 11451, KSA; ^3^ Department of Comparative Medicine, King Faisal Specialist Hospital and Research Center, Riyadh 11211, KSA; ^4^ Department of Pathology, King Faisal Specialist Hospital and Research Center, Riyadh 11211, KSA

**Keywords:** breast cancer, DNMT1, IL-6, mRNA decay, cancer-associated fibroblasts

## Abstract

The activation of breast stromal fibroblasts is a crucial step toward tumor growth and spread. Therefore, it is extremely important to understand the molecular basis of this activation and determine the molecules and the mechanisms responsible for its sustainability. In the present report we have shown that the DNA methyl-transferase protein DNMT1 is critical for the activation of breast stromal fibroblasts as well as the persistence of their active status. Indeed, we have first revealed DNMT1 up-regulation in most cancer-associated fibroblasts relative to their corresponding adjacent normal fibroblasts. This effect resulted from HuR-dependent stabilization of the *DNMT1* mRNA. Furthermore, ectopic expression of DNMT1 activated primary normal breast fibroblasts and promoted their pro-carcinogenic effects, both *in vitro* and in orthotopic tumor xenografts. By contrast, specific DNMT1 knockdown normalized breast myofibroblasts and repressed their cancer-promoting properties. These effects were sustained through inhibition of the IL-6/STAT3/NF-κB epigenetic cancer/inflammation positive feedback loop. Furthermore, we have shown that DNMT1-related activation of breast fibroblasts is mediated through upregulation of the RNA binding protein AUF1, which is also part of the loop. The present data demonstrate the critical function of DNMT1 in breast cancer-related sustained activation of breast stromal fibroblasts.

## INTRODUCTION

Breast cancer is a major health problem that threatens the lives of millions of women worldwide [1]. When cancer develops, the stroma adapts and coevolves to support the “function” of the tumor. Large amount of data indicate that cancer-associated fibroblasts (CAFs), constitute a substantial volume of the tumor stroma and play a pivotal role in tumor maintenance, dissemination, and even drug resistance [2, 3]. Orimo et al. have shown that CAFs promote tumor growth and angiogenesis through elevated SDF-1 secretion [4]. Several other cytokines, chemokines, growth factors and matrix metalloproteinases (MMPs) are secreted by stromal fibroblasts and their secretion increases when these fibroblasts are activated [2, 3]. Importantly, the active status of cancer-associated fibroblasts is persistent even in absence of cancer cells and after prolonged cell culture *in vitro* [4, 5]. We have recently shown that the sustained active pro-carcinogenic effects of CAFs is mediated through the stimulation of the positive IL-6/STAT3/NF-κB feedback loop [6]. This loop maintains the epigenetic transformed state of cells for many generations in the absence of the inducing signal. It has been also reported that this epigenetic switch is required for the self-renewing capacity of cancer stem cells [7]. In addition, different genetic and/or epigenetic alterations may also be involved. Indeed, several reports have previously shown that methylation plays an important role in myofibroblast formation [8, 9]. DNA methylation is a common early event in carcinogenesis, which is typically mediated by DNA methyltransferases (DNMTs) [10, 11]. DNMT1 is an important methyltransferase that maintains methylation during cell proliferation. Furthermore, DNMT1 has *de novo* activity in human cancer cells and plays also important roles in maintaining genome stability [12, 13]. It has been recently shown that activation of fibroblasts is sustained by constitutive activation of JAK1/STAT3 through a DNMT1-dependent down-regulation of SHP-1 [9]. DNMT1 is also indispensable for mammary and cancer stem cell maintenance and tumorigenesis [14]. Furthermore, the expression of DNMT1 was found to be higher in triple negative breast cancer and lower in luminal A samples [15]. These results and others have shown that DNMT1 plays crucial roles in breast carcinogenesis. Since breast stromal fibroblasts are also major players in this complex process, we sought to investigate the role of DNMT1 in these mesenchymal cells.

## RESULTS

### DNMT1 is upregulated in breast cancer-associated fibroblasts

**Figure 1:**
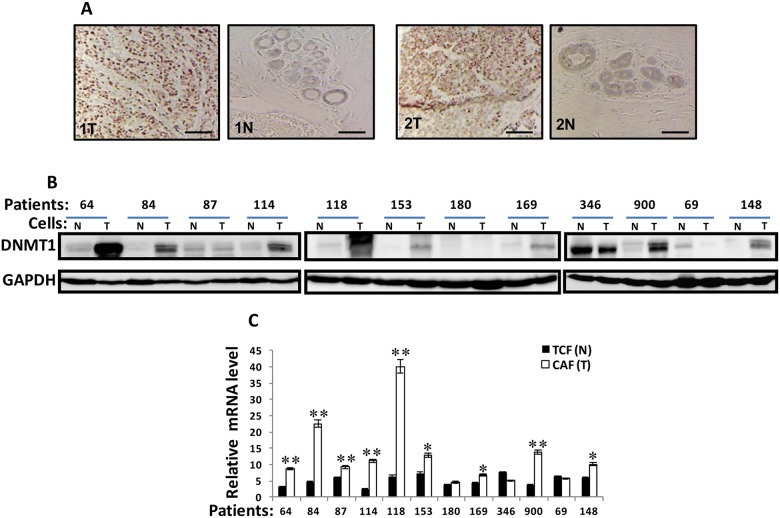
DNMTl is upregulated in CAFs versus TCFs **(A)** Formalin-fixed paraffin-embedded sections of breast resection specimens from 10 patients encompassing both invasive ductal carcinoma and their corresponding histologically normal adjacent tissues were utilized for immunohistochemical analysis using anti-DNMTI antibody. Zeiss Axio microscope was utilized, scale bar = 50 μM. **(B)** Whole-cell lysates were prepared from the indicated cells and were used for immunoblotting analysis, CAFs (T), TCFs (N). GAPDH was used as internal control. **(C)**. Total RNA was extracted and the level of the *DNMT1* mRNA was assessed by qRT-PCR. Error bars represent mean ± S.D (n=3). ^*^, *P*< 0.05; ^**^, *P*<0.001.

To address the role of DNMT1 in BSFs, we first assessed the level of DNMT1 in breast cancer tissues. Therefore, 10 pairs of paraffin embedded sections including both tumor and their adjacent normal tissues derived from patients suffering invasive ductal carcinoma of different stages and subtypes were immunostained by anti-DNMT1 antibody. The DNMT1 staining intensity was higher in tumors than in their paired normal tissues for both tumors and stromal cells (Figure 1A). To confirm this, and show the increase in DNMT1 level in CAFs, DNMT1 expression was assessed in 12 human breast CAFs and their counterpart fibroblasts isolated from adjacent histologicaly normal tumor tissues (TCFs). CAF/TCF pairs were always used simultaneously at similar passages, and exhibited similar cell cycle distribution as shown for the CAF/TCF-64 pair (Supplementary Figure 1). Whole cell extracts were prepared and specific anti-DNMT1 and anti-GAPDH (used as internal control) antibodies were utilized for immunoblotting analysis. Figure 1B shows that the level of the DNMT1 protein was higher in 8 out of 12 CAFs (67%) compared to their corresponding TCFs. However, DNMT1 level was similar in 2 CAF/TCF pairs 180 and 87, while it was lower in CAF-346 and CAF-69 compared to their paired TCF cells (Figure 1B). In addition, a great inter-individual variation in DNMT1 expression was observed between the various CAFs and also the different TCFs (Figure 1B).

The level of the *DNMT1* mRNA was also assessed in the same cells by quantitative RT-PCR (qRT-PCR). Figure 1C shows a clear increase in the *DNMT1* mRNA level in 9 out of 12 (75%) CAFs, compared to their adjacent TCFs, while *DNMT1* mRNA level was similar in the 2 CAF/TCF pairs 180 and 69, and it was reduced in CAF-364 relative to TCF-364. This shows that the expression of the *DNMT1* mRNA reflects that of the corresponding protein in the majority of the TCF/CAF pairs (Figure 1B and 1C), indicating that DNMT1 up-regulation is due, at least in part, to an increase in the level of its corresponding transcript.

### The DNMT1 mRNA decay is slower in CAFs versus TCFs

**Figure 2:**
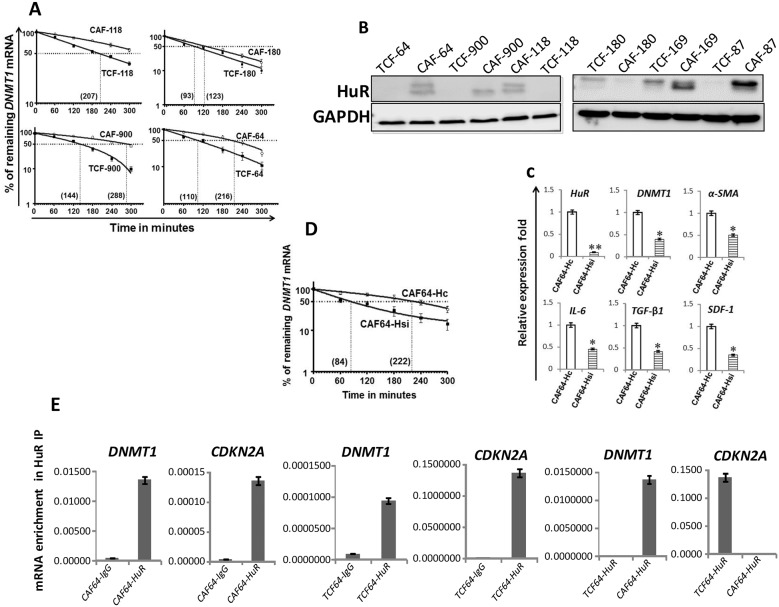
DNMTl upregulation in CAFs is mediated through HuR-dependent stabilization **(A)** and **(D)** Exponentially growing cells were treated with actinomycin D (5 μg/ml) for various periods of time, and total RNA was extracted and the level of the *DNMT1* mRNA was assessed using qRT-PCR. The values were determined and normalized against GAPDH. Graphs show the proportion of the *DNMT1* mRNA remaining post-treatment, and the dotted lines indicate the *DNMT1* mRNA half life, the corresponding values are in brackets. Error bars indicate mean ± S.D (n=3). **(B)** Whole-cell lysates were prepared from the indicated cells, and used for imrnunoblotting analysis. **(C)** Total RNA was extracted from CAF-64 cells expressing either HuR-siRNA (CAF64-Hsi) or control siRNA (CAF64-Hc), and the levels of the indicated genes were assessed by qRT-PCR and normalized against GAPDH. Error bars represent mean ± S.D. ^*^, *P*< 0.05; ^**^, *P*= 0.00066. **(E)** Whole cell lysates were prepared from CAF/TCF-64 cells, and then RNAs bound to the HuR protein were isolated by immunoprecipitation using anti-HuR antibody (CAF64-HuR and TCF64-HuR, respectively) or anti-lgG (CAF64-IgG and TCF64-IgG, respectively), and then the mRNAs of the indicated genes were amplified by qRT-PCR. Data were normalized to the levels of the highly abundant GAPDH mRNA in each IP sample and represented as the enrichment of each mRNA. Error bars indicate mean ± S.D (n=3).

In order to elucidate the molecular mechanism that underlies the *DNMT1* mRNA up-regulation in CAFs, we studied the *DNMT1* mRNA half-life in the TCF/CAF-64, 118, 900 and 180 pairs. Figure 2A shows that, while the *DNMT1* mRNA half-life reached 3 h 35 min in CAF-64, it was only 100 min in TCF-64. Similar results were obtained for 2 other CAF/TCF pairs (118 and 900) although with different DNMT1 half- lives (Figure 2A). On the other hand, the *DNMT1* mRNA half-life was similar in CAF-180 and TCF-180 (Figure 2A), which were shown to express similar levels of the *DNMT1* mRNA and protein (Figure 1A and 1B). These results indicate that the higher level of the *DNMT1* mRNA in CAFs is owing to a decrease in its turnover.

### DNMT1 stabilization in CAFs is HuR-dependent

To explore the molecular basis of *DNMT1* mRNA stabilization in CAFs relative to TCFs, we investigated the possible implication of the mRNA stabilizing protein HuR in DNMT1 upregulation in CAF cells. Figure 2B shows that the HuR protein is clearly upregulated in 5 CAFs compared to their corresponding TCFs. However, HuR level was rather slightly reduced in CAF-180 as compared to TCF-180, which parallels the reduced level of DNMT1 in CAF-180 relative to TCF-180. Importantly, the *DNMT1* mRNA was significantly up-regulated in all CAFs wherein HuR was found highly expressed (Figure 1C and 2B).

To further elucidate the link between HuR and DNMT1, we tested the effect of HuR knock-down using specific siRNA on the expression level of the *DNMT1* mRNA in CAF-64. Therefore, these cells were transfected with either HuR-siRNA (CAF64-Hsi) or a scrambled sequence (CAF64-Hc) for 48 h, and then cells were harvested. The *DNMT1* mRNA level was reduced more than 2 fold upon HuR silencing (Figure 2C). Similarly, the mRNA levels of *α-SMA*, *IL-6*, *TGF-β1* and *SDF-1* were also reduced in HuR-deficient cells compared to controls (Figure 2C). Furthermore, substantial decrease in the *DNMT1* transcript half-life was shown in HuR-deficient cells relative to controls (Figure 2D). To test the binding of HuR to the DNTM1 transcript, HuR-mRNAs ribonucleoprotein complexes were pulled-down from TCF-64/CAF-64 cells by immunoprecipitation (IP) using anti-HuR antibody (IgG was used as control), and the level of HuR bound mRNAs were assessed upon qRT-PCR amplifications. Figure 2E shows significant amplification of the *DNMT1* and *CDKN2A* (used as positive control) mRNAs following immunoprecipitation with anti-HuR, but not with the IgG antibody, indicating the binding of the HuR protein to these transcripts. Importantly, the level of the immunoprecipitated *DNMT1* mRNA was higher in CAF-64 compared to TCF-64 cells. However, the level of the *CDKN2A* mRNA associated to HuR was reduced in CAF-64 compared to TCF-64 cells (Figure 2E). Together, these results indicate HuR-dependent stabilization of the *DNMT1* mRNA in CAF cells.

### Ectopic expression of DNMT1 activates breast stromal fibroblasts

**Figure 3:**
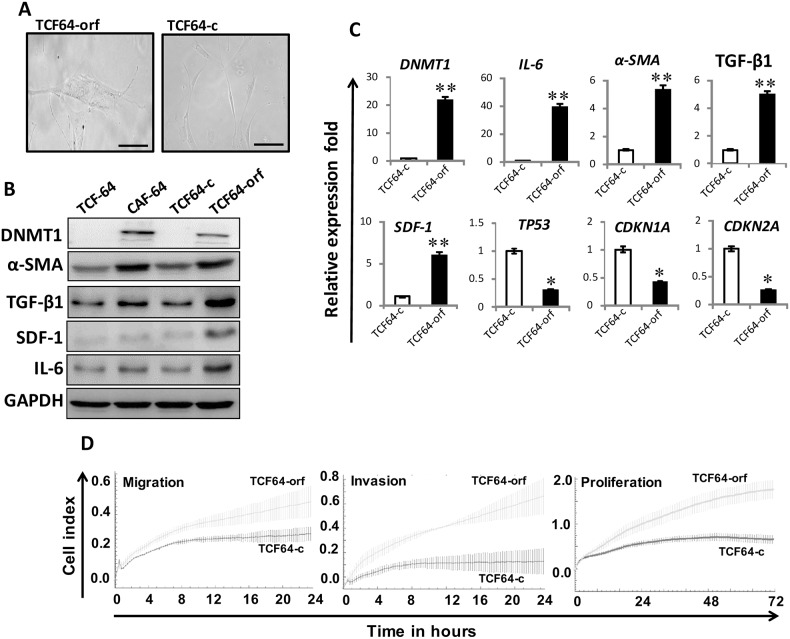
Ectopic expression of *DNMT1* activates breast stromal fibroblasts TCF-64 cells were transfected with a plasmid bearing the *DNMT1* ORF (TCF64-orf) or an empty vector (TCF64-c). **(A)** Phase contrast images of cells several days post-transfection. Fluid Cell Imaging Station was utilized, scale bar = 50 μM. **(B)** Whole-cell lysates were prepared from the indicated cells, and were used for immunoblotting analysis using antibodies against the indicated proteins. **(C)** Total RNA was extracted and the mRNA levels of the indicated genes were assessed using qRT-PCR. Error bars represent mean ± S.D (n=3). ^*^, *P*<0.05; ^**^, *P<*0.003. **(D)** Exponentially growing cells (10^4^) were added independently into the 16-well CIM-plates (invasion/migration) or to the E-plate (proliferation), and cell invasion, migration and proliferation were assessed using the RTCA-DP xCELLigence System. Data are representative of different experiments performed in triplicate.

To delineate the role of DNMT1 up-regulation in the activation of breast stromal fibroblasts, we introduced *DNMT1*-ORF or an empty vector into TCF-64 cells (TCF64-orf and TCF64-c, respectively). TCF64-orf and TCF64-c cells showed similar cell cycle distribution with 86% and 82% of cells at G0/G1, respectively (Supplementary Figure 1). Figure 3A shows that TCF64-orf cells acquired active fibroblast morphology (large and flat), while TCF64-c cells exhibited typical normal fusiform fibroblast morphology. Interestingly, the DNMT1 protein level increased in TCF64-orf compared to TCF64-c cells, which showed similarities with CAF-64 and TCF-64 cells (Figure 3B). Interestingly, like in CAF-64 cells, DNMT1 upregulation was accompanied by strong increase in the levels of α-SMA, SDF-1, TGFβ-1 and IL-6 (Figure 3B). Similar effects were observed at the mRNA levels of these genes (Figure 3C). On the other hand, the levels of the *TP53*, *CDKN1A* and *CDKN2A* mRNAs were reduced upon DNMT1 up-regulation (Figure 3C). Likewise, ectopic expression of *DNMT1* in normal breast fibroblasts (NBF-11) upregulated α-SMA, SDF-1, IL-6, TGF-β1 at both the mRNA and protein levels, and activated AKT and STAT3 as compared to control cells (Supplementary Figure 2). Furthermore, DNMT1 upregulation repressed p53, p21 and p16 (Supplementary Figure 2B), and enhanced the migration, invasion and proliferation abilities of TCF64 cells (Figure 3D). This suggests that DNMT1 up-regulation activates BSFs.

### DNMT1 upregulation enhances the paracrine procarcinogenic effects of breast stromal fibroblasts

**Figure 4:**
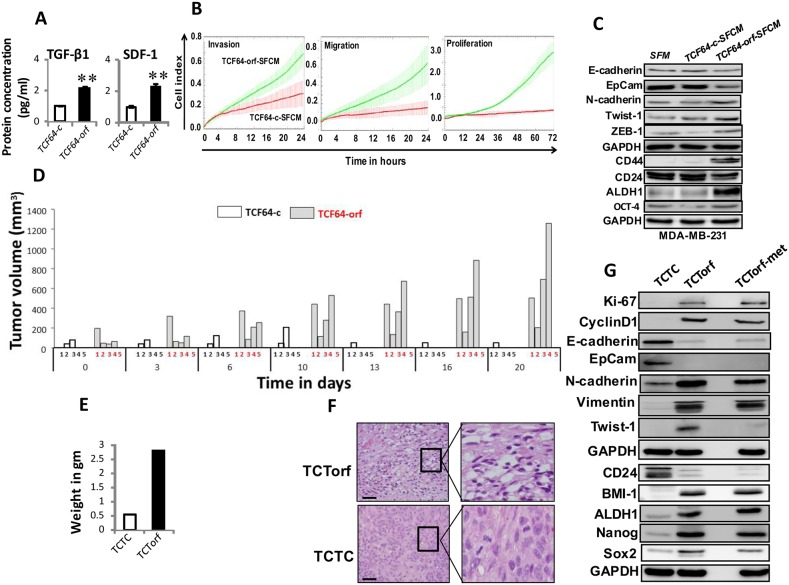
Ectopic expression of DNMT1 enhances the paracrine procarcinogenic effects of breast stromal fibroblasts *in vitro* and *in vivo* **(A)** SFCM from the indicated cells were collected after 24 h and the levels of the indicated proteins were determined by ELISA and were presented in the respective histograms. Error bars indicate mean±S.D. ^**^, *P*<0.001 (n=3). **(B)** MDA-MB-231 cells were seeded in the presence of SFCM from TCF64-orf (TCF64-orf-SFCM) or TCF64-c (TCF64-c-SFCM) cells, and then the migration, invasion and proliferation abilities were assessed by the real time RTCA-DP xCELLigence System. Data are representative of different experiments performed in triplicate. **(C)** Whole cell lysates were prepared from MDA-MB-231 cells treated as indicated and were used for immunoblotting using the indicated antibodies. **(D)** Orthotopic breast cancer xenografts were created by co-injecting MDA-MB-231 cells with TCF64-orf or TCF64-c cells (n=5) under the nipple of nude mice. The histogram shows the volumes of xenograft tumors that were measured at the indicated time points. **(E)** Orthotopic tumors bearing TCF64-orf cells (TCTorf) or TCF64-c cells (TCTC) were excised and weighted. **(F)** Excised tissues were subjected to hematoxylin and eosin staining. Olympus BX53 microscope was utilized, low magnification (left); scale bar = 25 μM. High maginification (right). **(G)** TCTorf, TCF64-c as well as metastatic (TCTorf-met) tumors were excised and whole cell lysates were prepared and protein levels were assessed by immunoblotting using antibodies against the indicated proteins.

Next, we sought to investigate the paracrine effects of BSFs that express high levels of DNMT1 on breast cancer cells. To this end, TCF64-orf and TCF64-c cells were cultured in serum-free medium (SFM) for 24 h, and then serum-free conditioned medium (SFCM) from each culture was collected and applied to ELISA test, which showed that the levels of secreted SDF-1 and TGFβ-1 increased more than 2 fold in TCF64-orf compared to controls (Figure 4A). Subsequently, we tested the paracrine effect of TCF64-orf cells on MDA-MB-231 cells. The migration, invasion and proliferation capacities were higher in the presence of TCF64-orf-SFCM than in the presence of TCF64-c-SFCM (Figure 4B). Figure 4C shows that TCF64-orf-SFCM decreased the level of the E-cadherin and EpCAM, while it upregulated the mesenchymal markers N-cadherin, ZEB-1 and Twist-1 relative to their levels in control cells. This indicates that BSFs that express high level of DNMT1 can paracrinally enhance the EMT process in breast cancer cells. Thereby, we tested the possible induction of stemness in these cells in a paracrine manner. Indeed, TCF64-orf-SFCM increased the expression of the 3 stemness markers, namely CD44 and ALDH1 compared to control cells, while it inhibited the level of CD24 (Figure 4C). TCF64-orf-SFCM also upregulated the pluripotency protein OCT-4 (Figure 4C). These results demonstrate the pro-carcinogenic effects of breast stromal fibroblasts that express high level of DNMT1.

### Ectopic expression of DNMT1 in fibroblasts enhances their pro-carcinogenic effects *in vivo*

Next, orthotopic breast cancer xenografts were created by co-injecting MDA-MB-231 cells with TCF64-orf (TCTorf) or TCF64-c (TCTC) cells (n=5/each group) under the nipple of nude mice. Interestingly, tumors containing TCF64-orf appeared only 10 days post-injection on 4 out of 5 mice and grew faster than those containing TCF64-c, which appeared after 2 weeks, on only 2 out of 5 animals (Figure 4D). Notably, one out of 4 mice bearing tumor containing TCTorf developed metastasis under the shoulder (TCTorf-met). Tumors were then excised and a significant difference in tumor weight was observed (Figure 4E). Hematoxylin and eosin staining showed higher number of mitotic and necrotic cells in TCTorf as compared to TCTC (Figure 4F). Furthermore, TCTorf as well as TCTorf-met expressed high level of the 2 proliferative markers Ki-67 and cyclin D1 (Figure 4G). Moreover, the levels of the epithelial markers E-cadherin and EpCam were markedly lower in TCTorf and TCTorf-met than in TCTC, while the levels of the mesenchymal markers N-cadherin and vimentin were much higher in TCTorf and TCTorf-met than in TCTC, while Twist-1 level increased only in TCTorf but not in TCTorf-met (Figure 4G). Therefore, TCF64-orf cells induced EMT *in vivo* as well. Additionally, TCTorf and TCTorf-met expressed high levels of the 4 stemness markers BMI-1, ALDH1, Nanog and Sox-2, but lower levels of CD24 as compared to TCTC (Figure 4G). The fact that the anti-Sox-2, anti-N-cadherin and anti-ALDH1 antibodies are human specific confirmed the human nature of the metastatic tumors. These results indicate that *DNMT1* upregulation in BSFs promotes breast carcinogenesis.

### DNMT1 knockdown suppresses active breast stromal fibroblasts

**Figure 5:**
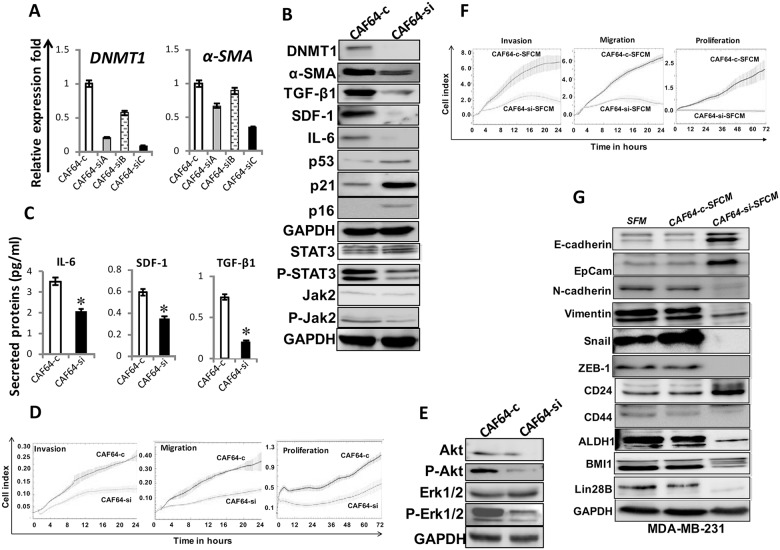
DNMT1 downregulation suppresses myofibroblasts CAF-64 cells were transfected with 3 different *DNMT1-siRNA* sequences (A), (B) and (C) (CAF64-si) and a scrambled sequence was used as control (CAF64-c). **(A)** Total RNA was extracted and used for qRT-PCR. Error bars represent mean ± S.D. **(B)** and **(E)** Whole-cell lysates were prepared from CAF64si and CAF64-c, and then were used for immunoblotting analysis using specific antibodies against the indicated proteins. **(C)** SFCM from the indicated cells were collected after 24 h and the levels of the indicated proteins were determined by ELISA and were presented in the respective histograms. Error bars indicate mean ± S.D (n=3). ^*^, *P*<0.05. **(D)** Exponentially growing cells were seeded, and cell invasion, migration and proliferation were assessed using the RTCA-DP xCELLigence System. Data are representative of different experiments performed in triplicate. **(F)** MDA-MB-231 cells were seeded in the presence of SFCM from CAF64-si (CAF64-si-SFCM) or CAF64-c (CAF64-c-SFCM) cells, and then the migration, invasion and proliferation abilities were assessed by the real time RTCA-DP xCELLigence System. Data are representative of different experiments performed in triplicate. **(G)** Whole cell lysates were prepared from MDA-MB-231 cells treated as indicated and were used for immunoblotting using the indicated antibodies.

Next, CAF-64 cells were transfected with either DNMT1 siRNA (3 variants A, B and C) or a scrambled sequence that was used as control. Figure 5A shows that DNMT1-si**C** caused the most significant reduction in the expression of both *DNMT1* and *α-SMA* mRNAs. DNMT1 protein level declined 8 fold in the DNMT1-siRNA-C treated cells (CAF64-si) compared to controls (CAF64-c) (Figure 5B). Interestingly, DNMT1 knock-down did not affect cell cycle distribution of cells (Supplementary Figure 1). Moreover, DNMT1 down-regulation reduced the protein level of α-SMA, SDF-1, TGF-β1, and IL-6, and inhibited the JAK2/STAT3 pathway (Figure 5B). However, p16, p21 and p53 levels were markedly upregulated upon DNMT1 knockdown in CAF-64 (Figure 5B). Similar effect was obtained at their mRNA levels (Supplementary Figure 3). In addition, ELISA showed that DNMT1-downregulation reduced the secretion of IL-6, SDF-1 and TGF-β1 (Figure 5C). Figure 5D shows that CAF64-c cells exhibited higher migration, invasion and proliferation abilities than CAF64-si cells. Moreover, CAF64-si cells expressed low levels of Akt/P-Akt and Erk1/2/P-Erk1/2 compared to CAF64-c cells (Figure 5E). This indicates that DNMT1 knockdown restrains the secretion of several cancer-promoting proteins and inhibits the migration/invasion/proliferation abilities of BSFs.

### DNMT1 knockdown represses the cancer-promoting effects of breast myofibroblasts

Figure 5F shows that the migration, invasion and proliferation capacities of MDA-MB-231 cells were lower in the presence of CAF64-si-SFCM than in the presence of CAF64-c-SFCM. Furthermore, CAF64-si-SFCM decreased the level of the mesenchymal markers N-cadherin, ZEB-1, Snail and vimentin, while it upregulated the epithelial markers E-cadherin and EpCam compared to control cells (Figure 5G). In addition, CAF64-si-SFCM inhibited the stemness properties in breast cancer cells (CD44^high^/CD24^low^/ALDH^high^), and downregulated BMI-1 and lin-28B compared to controls (Figure 5G). This indicates that DNMT1 downregulation inhibits the paracrine pro-carcinogenic effects of breast myofibroblasts.

### DNMT1 mediates IL-6-dependent activation of breast stromal fibroblasts

**Figure 6:**
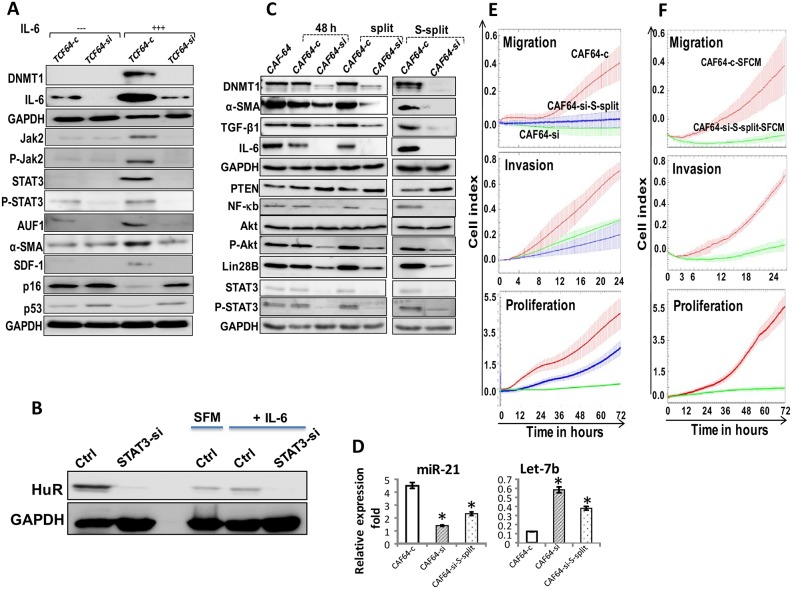
DNMTl knockdown persistently inhibits the pro-inflammatory/carcinogenic IL-6-related loop and CAF cells **(A)** TCF64-si and TCF64-c cells were either sham-treated or exposed to IL-6 (35 ng/mL), and then whole cell lysates were prepared and were used for immunoblotting using specific antibodies for the indicated proteins. **(B)** TCF-64 cells were transfected with STAT3-siRNA (STAT3si) or control siRNA (Ctrl), and then cells were either sham-treated or exposed to IL-6 (3.5 ng/mL), and then the expression of the HuR protein was assessed by immunoblotting. **(C)** CAF64-si and CAF64-c cells were cultured for 48 h, and then the medium-containing DNMTl-siRNA or a scrambled sequence was removed and replaced with fresh medium for 48 h (48 h). Cells were splited (split), and then splited 6 more times (S-split). Whole-cell lysates were prepared from the indicated cells and were used for immunoblotting analysis using specific antibodies against the indicated proteins. **(D)** Total RNA was extracted, and *U6* was used as internal control and the mature miRNA levels were assessed using qRT-PCR. Error bars represent mean ± S.D (n=3). ^*^, *P*<0.02. **(E)** Migration, invasion and proliferation abilities of the indicated cells were analyzed by the real time RTCA-DP xCELLigence System. **(F)** MDA-MB-231 cells were seeded with the indicated SFCM, and then migration, invasion and proliferation abilities were analyzed by the real time RTCA-DP xCELLigence System. Data are representative of different experiments performed in triplicate.

We have recently shown that IL-6 can activate breast stromal fibroblasts through the activation of the JAK2/STAT3 pathway and the consequent up-regulation of AUF1 [16]. Therefore, we sought to investigate the possible implication of DNMT1 in this IL-6-related BSF trans-differentiation. To this end, TCF64-si and TCF64-c cells were either sham-treated or exposed to recombinant IL-6 (3.5 ng/mL). Figure 6A shows that IL-6 increased the level of DNMT1 as well as IL-6 only in TCF64-c but not in DNMT1-deficient cells (TCF64-si). Similarly, DNMT1 knockdown abolished IL-6-dependent activation of STAT3, AUF1 and their targets α-SMA, SDF-1, and also the downregulation of p16 and p53, which are hallmarks of active breast stromal fibroblasts (Figure 6A). This indicates that IL-6 upregulates DNMT1, which plays important role in IL-6-dependent activation of breast stromal fibroblasts.

Since DNMT1 is under the control of HuR, we sought to investigate the role of STAT3 in the expression of HuR, and its induction in response to IL-6. Therefore, STAT3 was knocked-down in TCF-64 cells transfected with either STAT3-siRNA (STAT3-si) or control siRNA (Ctrl). Figure 6B shows that STAT3 down-regulation reduced the level of HuR protein. This indicates that STAT3 modulates the expression of the HuR gene in BSFs. Subsequently, STAT3si and Ctrl cells were treated with exogenous recombinant IL-6 or SFM (used as negative control) for 24 h. Figure 6B shows that STAT3 knockdown abolished IL-6-dependent upregulation of HuR. This indicates that the IL-6-related upregulation of HuR is STAT3-dependent.

### DNMT1 knockdown persistently suppresses the proinflammatory/carcinogenic IL-6- related loop and inhibits breast myofibroblasts

We have recently shown that IL-6 permanently activates BSFs through the activation of the IL-6 positive feedback loop [6]. Since IL-6 up-regulates DNMT1, we sought to check the possible persistent effect of DNMT1 downregulation. Therefore, CAF64-si and CAF64-c cells were incubated for 48 h post-transfection in fresh medium (48 h), and then were split once (split) followed by 6 times spliting (S-split). Figure 6C and 6D shows that DNMT1 remained down-regulated despite transient knockdown of the gene using siRNA and following several cellular splitting. Similarly, IL-6 and other members of the IL-6/STAT3/NF-κB autocrine positive feedback loop such as NF-κB (p65), Akt/P-Akt, STAT3/P-STAT3, Lin-28B and miR-21 remained decreased/inhibited in CAF64-si cells compared to CAF64-c cells. By contrast, the level of PTEN and Let-7b remained up-regulated in CAF64-si cells as compared to controls (Figure 6C and 6D). These results suggest that the transient DNMT1 down-regulation permanently inhibited the IL-6-related positive feedback loop. Since this loop is implicated in the sustained active status of BSFs [6], we investigated whether transient DNMT1 down-regulation has persistent inhibitory effect on myofibroblasts. Indeed, Figure 6C shows sustained reduced levels of α-SMA and TGF-β1 in CAF64-si compared to CAF64-c, even after several splits. This was confirmed by showing that CAF64-si-S-split cells had reduced migration/invasion as well as proliferation capacities compared to control cells (Figure 6E). Furthermore, SFCM from these cells suppressed the migration/invasion and proliferation abilities of breast cancer MDA-MB-231 cells in a paracrine manner (Figure 6F). These results indicate that DNMT1 is part of the IL-6/STAT3/NF-κB autocrine positive feedback loop, and that transient inhibition of this gene leads to persistent inhibition of breast myofibroblasts and their pro-carcinogenic effects.

### DNMT1 effects in breast stromal fibroblasts are AUF1-dependent

**Figure 7:**
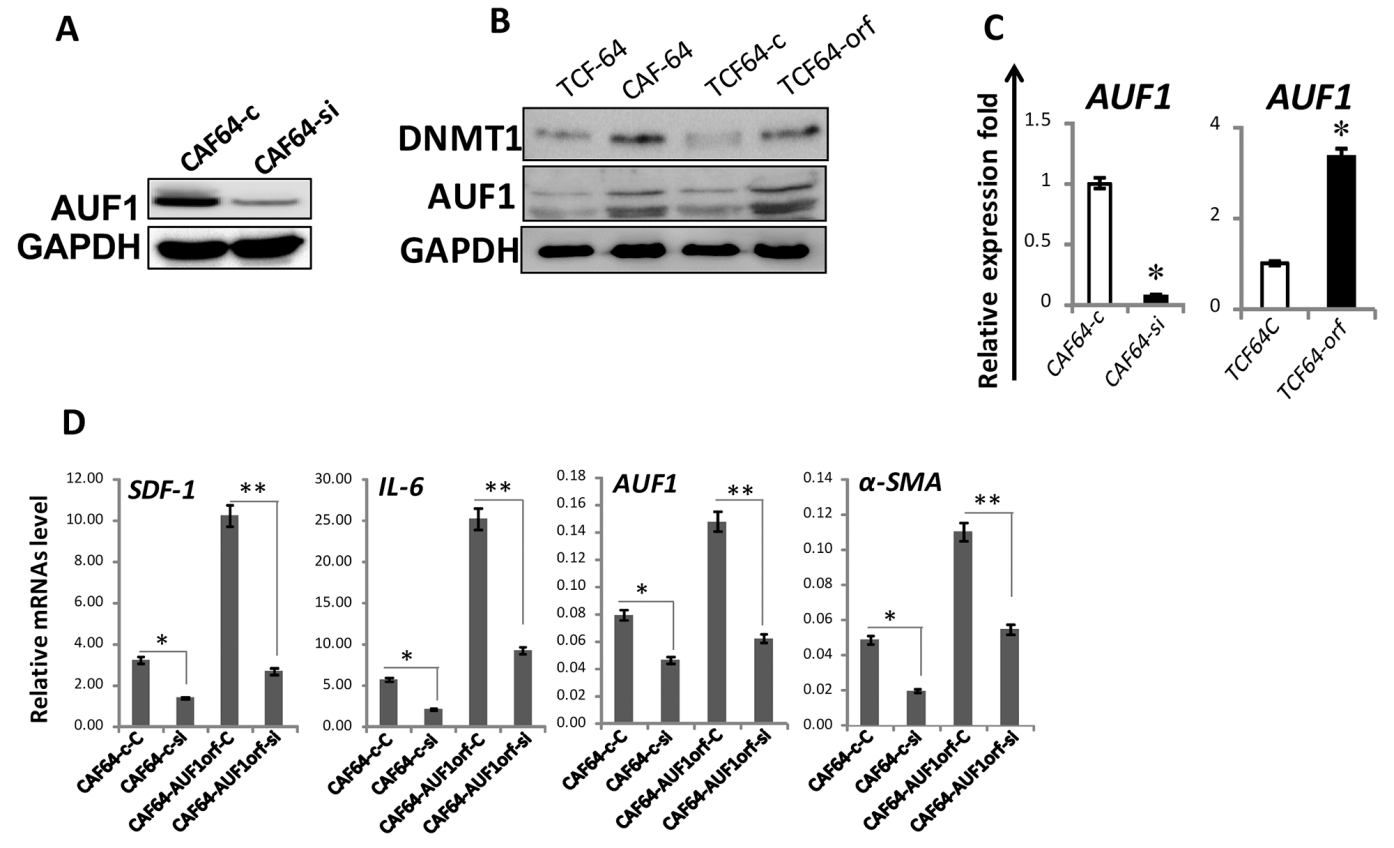
DNMTl effects in breast stromal fibroblasts are AUF1-dependent **(A)** and **(B)** Immunoblotting, legends are as in Figure 5B and 3B, respectively. **(C)** Total RNA was purified from the indicated cells, and the AUF1 mRNA level was assessed by qRT-PCR. Error bars represent means ± S.D (n=3). ^*^, *P*< 0.03. **(D)** AUF1-orf or an empty vector were introduced into CAF-64 cells (CAF64-AUF1orf, CAF64-c, respectively), after 48 h, the media were removed and replaced with fresh complete medium for another 24 h, and then cells were transfected with DNMTl-siRNA or a scrambled sequence for 48 h (CAF64-AUF1orf-si, CAF64-AUF1orf-C and CAF64-c-C, CAF64-c-si, respectively). Total RNA was purified and the mRNA level of the indicated genes were assessed in the indicated cells using qRT-PCR. Error bars represent mean ± S.D (n=3). ^*^, *P*<0.05; ^**^, *P*<0.01.

The obtained data raised an important question as to how DNMT1 activate BSFs. Since AUF1 plays a major role in this process, we first tested the effect of DNMT1 on AUF1 expression. Figure 7A and 7B shows that while DNMT1 down-regulation reduced the level of AUF1, DNMT1 ectopic expression upregulated AUF1. Similar results were obtained at the mRNA level (Figure 7C), and also in NBF-11 cells expressing DNMT1- ORF (NBF11-orf) as compared to control cells (NBF11-c) (Supplementary Figure 4). This indicates that AUF1 expression is modulated in a DNMT1-dependent manner.

We have next asked whether AUF1 plays a role in DNMT1-dependent activation of breast stromal fibroblasts. To study this, we investigated the effect of ectopic expression of AUF1 in DNMT1-deficient cells on the expression of *α-SMA, SDF-1* and *IL-6* by qRT-PCR. Figure 7D shows that the ectopic expression of AUF1 enhanced the level of these mRNAs in CAF64-si cells (DNMT1-deficient), and therefore eliminated the effect of DNMT1 deficiency, restoring the expression levels observed in control cells (CAF64-c). This indicates that the effect of DNMT1 on *α-SMA*, SDF-1 and IL-6 is mediated through AUF1.

## DISCUSSION

In the present report, we have first shown that DNMTI is upregulated in CAFs relative to their adjacent TCFs, at both the mRNA and protein levels. The *DNMT1* mRNA was more stable in different CAFs relative to their corresponding TCFs, suggesting the role of cancer cells in slowing-down the *DNMT1* mRNA turnover leading to its accumulation in the surrounding fibroblasts. This stabilization of the *DNMT1* mRNA is mediated through HuR, which binds the *DNMT1* mRNA and reduces its turnover. Similarly, Lopez de Silanes *et al.* showed that HuR binds and stabilizes the *DNMT3b* mRNA in human colorectal cancer cells [17]. This indicates that HuR may play major roles in methylation modulation through controlling the expression of 2 important methylation proteins DNMT1 and DNMT3b. Interestingly, HuR was up-regulated in most CAFs relative to their corresponding TCFs, with great correlation with the *DNMT1* mRNA level (Figure 2B and 1C). Furthermore, HuR knock-down repressed the 4 major myofibroblast markers α-SMA, IL-6, TGF-β1 and SDF-1, confirming the importance of HuR in the activation of BSFs. HuR is a well known RNA binding protein, which stabilizes the message of a plethora of genes involved in various physiological processes, including carcinogenesis [18, 19]. HuR-dependent stabilization of DNMT1 in CAFs relative to TCFs, suggested an important role of this protein in the activation of BSFs during breast carcinogenesis. Indeed, ectopic expression of DNMT1 activated breast stromal fibroblasts and enhanced their pro-carcinogenic effects both *in vitro* and in orthotopic mice model of breast cancer, while specific DNMT1 knockdown suppressed breast myofibroblasts and inhibited their tumor-promoting capacities. DNMT1 up-regulation enhanced the expression of the major myofibroblast marker α– SMA, as well as SDF-1, TGF-β1 and IL-6, which play major roles in the autocrine and paracrine effects of breast stromal fibroblasts [2]. On the other hand, DNMT1 negatively regulated the 3 important tumor suppressor proteins p16, p2I and p53, which suppress breast fibroblasts [20, 21]. This suggests that DNMT1 may not directly regulate the expression of these genes through methylation, since some of them such as SDF-1, TGF-β1 and IL-6 were induced upon DNMT1 up-regulation. This confirms the methylation-independent function of DNMT1. Indeed, it has been previously shown that catalytically inactive DNMT1 represses more than 1000 genes in colon cancer cells [22]. Furthermore, approximately 30% of the upregulated genes in DNMT1 knockout cells do not contain dense CpG islands [23]. Thereby, since all these active fibroblast-related genes are under the control of the RNA binding protein AUF1, we tested the possible implication of AUF1 in DNMTl-related regulation of these genes. We have shown that DNMT1 positively controls the expression of AUF1, which mediates the regulatory effect of DNMT1 on these genes in breast stromal fibroblasts. This indicates that AUF1 is a key player in the activation of BSFs through regulating a plethora of important genes. As a case in point, we have previously shown that AUF1 is also up-regulated in most CAFs relative to their TCF counterparts [21].

**Figure 8:**
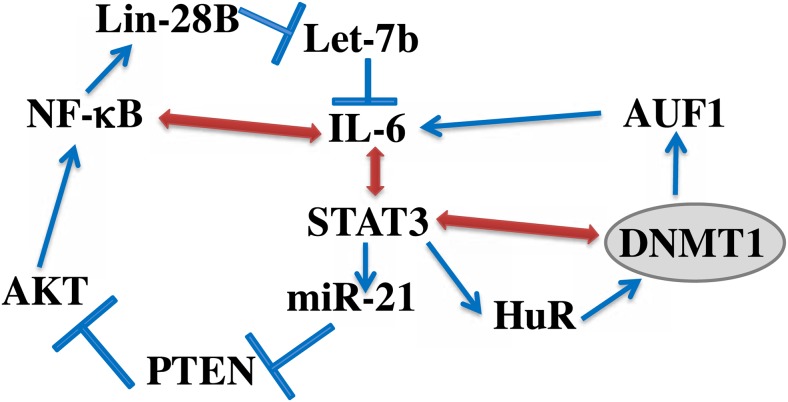
Schematic representation of the IL-6/STAT3/NF-κB feedback loop, and the implication of HuR and DNMT1 See text for more details.

Additionally, we have shown that exogenous recombinant IL-6, which has been previously shown to activate BSFs [16], up-regulates HuR and its downstream target DNMTl in a STAT3-dependent manner, and that this increase in DNMT1 level is required for the IL-6-dependent activation of BSFs. This further confirmed the important role of DNMT1 up-regulation in the activation of breast fibroblasts. Since AUF1 is part of the IL-6/STAT3 positive feedback loop, which sustains the active status of CAF cells even in absence of the activating factor [6], we tested the possible implication of DNMTl in this loop. Interestingly, transient DNMTl knockdown led to permanent reversion of BSFs, with sustained cellular and molecular effects of the transient DNMT1 downregulation even following several cell splitings as well as freezing/thawing processes. This suggests that DNMTl is also part of this epigenetic loop as summarized in Figure 8. It has been previously shown that DNMT1 is under the control of STAT3 [24], a major player in this loop [7, 25]. Since DNMTl is involved in the methylation process, it is also possible that this inflammation/cancer-related epigenetic loop is fueled either directly or indirectly by methylation processes, which helps maintaining the active status of myofibroblasts. Therefore, DNMT1 upregulation could maintain the active form of myofibroblasts through 2 epigenetic mechanisms, the IL-6/STAT3 positive feedback loop as well as methylation. In a recent report, it has been shown that combined inhibition of JAK/STAT signaling and DNMT activities by ruxolitinib or 5’-aza-2’-deoxycytidine results in sustained reversion of CAF-associated proinvasive activity [9]. However, in the present study, transient DNMTl downregulation by specific siRNA was sufficient to permanently inhibit the epigenetic loop as well as the invasion/migration and proliferation of active BSFs. Let-7b and miR-21 are also part of this loop, and while DNMTl induced let-7b, it rather suppressed miR-21, which confirms the methylation-independent functions of DNMT1. Since these 2 miRNAs control the expression of several genes, it's clear that DNMT1 may also regulate these genes through let-7b, miR-21 and may be other microRNAs.

Moreover, we have shown that DNMT1 activates STAT3, which suggests the presence of a positive feedback loop between STAT3 and DNMT1 (Figure 8). DNMT1-dependent activation of STAT3 could be mediated through JAK2 and IL-6 since DNMT1 activated both proteins. There is also physical association between STAT3 and DNMT1, which is crucial for DNA methylation [26, 27].

In summary, the present data indicate that DNMT1 plays major roles in the transactivation of breast stromal fibroblasts and the consequent cancer-promoting properties. We have also shown that the 2 important RNA binding proteins HuR and AUF1, are critical for these DNMT1-related processes, since HuR stabilizes *DNMT1*, while AUF1 is induced in a DNMT1-dependent manner. DNMT1 and AUF1 are members of the IL-6/STAT3 positive feedback loop, responsible for the sustained active status of breast myofibroblasts.

## MATERIALS AND METHODS

### Cells and cell culture

Breast fibroblast cells were obtained and used as previously described [5]. MDA-MB-231 cells were purchased in 2011 from ATCC and authenticated using short tandem repeat profiling by ATCC, propagated, expanded, and frozen immediately into numerous aliquots after arrival. The revived cells were utilized within 10 to 12 passages and not exceeding a period of 3 months. Cells were regularly screened for mycoplasma contamination using MycoAlert Mycoplasma Detection Kits (Lonza). All supplements were obtained from Sigma (Saint Louis, MO, USA) except for antibiotic and antimycotic solutions, which were obtained from gibco (Grand Island, NY, USA). Cells were maintained at 37°C in humidified incubator with 5% CO_2_.

### Cellular lysate preparation and immunoblotting

This has been performed as previously described [28]. Antibodies directed against DNMT1 (ab19905), alpha smooth muscle actin (α-SMA), Ki-67, transforming growth factor beta 1 (TGF-β1), Stromal-derived factor-1 (SDF-1), Twist-1, Vimentin (RV202), AUF1 (ab50692), N-cadherin and interleukin-6 (IL-6) were purchased from Abcam (Cambridge, MA); STAT3, pSTAT3-Tyr705 (D3A7), Snail (C15D3), E-cadherin (24E10), EpCam (UV1D9), JAK-2 (D2E12) and phospho-JAK-2 (TYR1007/1008), Cyclin D1 (2922), Akt, phospho-Akt (193H12), Nanog, NF-κB, OCT-4, PTEN, BMI-1, Lin-28B, Sox-2, ERK1/2 (137F5), phosph-ERK1/2 Glyceraldehydes-3-phosphate dehydrogenase (GAPDH, FL-335) from Cell Signaling (Danvers, MA); ZEB-1 (4C4) from Abnova (Taipei, Taiwan); p21 (F-5), p53 (DO-1), HuR (3A2), CD44 (Sp37) and CD24 (C-20) were purchased from Santa Cruz (Santa Cruz, CA), p16 (BD) was purchased from BD Biosciences (UK). Secondary antibodies are anti-Rabbit IgG HRP Conjugate (REF#W401), anti-mouse IgG HRP Conjugate (REF W402) and donkey anti-Goat IgG (H+L), HRP Conjugate (REF V805), from Promega (USA).

### RNA purification and qRT-PCR

Total RNA, containing miRNAs, was purified using the miRNeasy mini kit (Qiagen, UK) according to the manufacturer's instructions and was treated with RNase-free DNase. 1 μg RNA was used to synthesize cDNA utilizing either Advantage RT-PCR kit (Clontech Laboratories, Mountain View, CA, US) or miScript II RT kit (Qiagen, UK) for mature miRNAs. Quantitative RT-PCR was performed in triplicate using 4 μl cDNA mixed with 2x FastStart Essential DNA Green qPCR mastermix (Roche, New York, NY, US) and 0.3 μM forward and reverse primers. Amplifications were performed utilizing the LightCycler 96 Real-time PCR detection system (Roche) using the following cycle conditions: 95°C for 10 min (1 cycle); 95°C for 10 sec, 59°C for 20 sec, 72°C for 30 sec (45 cycles). GAPDH expression levels were used for normalization, and gene expression differences were calculated using the threshold cycle (Ct). Three independent experiments were performed for each reaction, and the obtained values were plotted as mean ± SD. The respective primers are:

*CDKN1A*: 5’-AGGTGGACCTGGAGACTCTC AG-3’ and 5’-TCCTCTTGGAGAAGATCAGCCG -3’;
*CDKN2A*: 5’-CTCGTGCTGATGCTACTGAG GA-3’ and 5’-GGTCGGCGCAGTTGGGCTCC -3’;
*TP53*: 5’-CCTCAGCATCTTATCCGAGTGG-3’ and 5’- TGGATGGTGGTACAGTCAGAG C -3’;
*GAPDH*: 5’-GAGTCCACTGGCGTCTTC-3’ and 5’-GGGGTGCTAAGCAGTTGGT-3’;
*SDF1*: 5’- CTCAACACTCCAAACTGTGCCC -3’ and 5’-CTCCAGGTACTCCTGAATCCAC-3’;
*ACTA2(α-SMA)*: 5′-CTATGCCTCTGGACGCAC AACT -3′ and 5′-CAGATCCAGACGCATGATGGCA -3′;
*TGF*β*1*: 5’-TACCTGAACCCGTGTTGCTCTC -3’ and 5’-GTTGCTGAGGTATCGCCAGGAA -3’;
*IL-6*: 5’-AGACAG CCA CTC ACC TCT TCA G-3’ and 5’- TTC TGC CAG TGC CTC TTT GCT G -3’;
*DNMT1*: 5’- AGGTGGAGAGTTATGACGAGGC -3’ and 5’-AGACAGCCACTCACCTCTTCAG-3’;
*AUF1*: 5’-GATCAAGGGGTTTTGGCTTT -3’ and 5’-GTTGTCCATGGGGACCTCTA-3’;
*ELAV-1(HuR)*: 5’-AGAGGTGATCAAAGACG CCA-3’ and 5’-ACTTCACTGTGATGGGCTCA-3’.

### Immunoprecipitation and qRT-PCR

Cell lysates were prepared from confluent cells, and 3 mg of proteins were incubated in the lysis buffer (50 mM Tris (pH 8), 100 mM NaCl, 10% glycerol, protease inhibitors, 5 mM DTT and 2 U/ml RNasin) and 4 μg of HuR mouse monoclonal antibody (mouse IgG was used as control) were added and mixed overnight at 4°C. Equal volume of protein A agarose was added per immunoprecipitation and mixed at 4°C for 2 h. After centrifugation, the pellet was re-suspended in 1 ml TRI reagent used for RNA extraction. qRT-PCR reactions were performed as described above.

### siRNA transfection

The transfections using DNMT1-siRNA (Origene, SR301244A), STAT3-siRNA (Qiagen, USA), HuR-siRNA (Metabion) and control–siRNA were carried out using the RNAi Fect reagent (Qiagen) as recommended by the manufacturer.

### Viral infection

Lentivirus-based vector bearing *DNMT1*–ORF as well as its corresponding control (GeneCopoeia) were used to prepare the lentiviral supernatant from 293FT cells. Lentiviral supernatants were collected 48 h post-transfection, filtered and used for infection. 48 h later, media were replaced with complete media and cells were grown for 3 days.

### DNMT1-ORF transfection

Lentivirus-based vectors bearing *DNMT1*-ORF and the corresponding control were used to carry out transfection of BSF cells using human dermal fibroblast nucleofector 2000 transfection kit (Invitrogen) following the manufacturer's recommendations. After 5 days, transfected cells were selected by puromycin (1μg/mL).

### Analysis of mRNA stability

Cells were challenged with Actinomycin D (5 μg/mL) for various periods of time (0-6 h), and then total RNA was purified and assessed using qRT-PCR. One-phase exponential decay curve analysis (Sigma Plot) was used to assess the mRNA decay kinetics, considering the values at time 0 as 100%. The time corresponding to 50% remaining mRNA was considered as mRNA half- life.

### ELISA assays

Supernatants from 24 h fibroblast cell cultures were harvested and ELISA was performed according to the manufacturer's instructions (R&D Systems). The OD was used at 450-nm on a standard ELISA plate-reader. These experiments were performed in triplicates, and were repeated several times.

### Cell migration, invasion and proliferation assays

This has been performed as previously described [29]. These assays were performed in a label-free real-time setting using the xCELLigence RTCA technology (Roche, Germany) that measures impedance changes in a meshwork of interdigitated gold microelectrodes located at the well bottom (E-plate) or at the bottom side of a micro-porous membrane (CIM plate 16). Cell migration and invasion were assessed as per the manufacturer's instructions. In brief, 2×10^4^ cells in serum-free medium were added to the upper wells of the CIM-plate coated with a thin layer of Matrigel (BD Biosciences) basement membrane matrix diluted 1:20 in serum-free medium (invasion) or non-coated (migration). Complete medium was used as a chemo-attractant in the lower chambers. Subsequently, the plates were incubated in the RTCA for 24 h and the impedance value of each well was automatically monitored by the xCELLigence system and expressed as Cell Index (CI) value, which represents cell status based on the measured electrical impedance change divided by a background value. Each assay was biologically performed in triplicate.

For the proliferation assay, exponentially growing cells (2×10^4^) were seeded in E-plate with complete medium as per the manufacturer's instruction. Cell proliferation was assessed for 48 h. All data were recorded and analyzed by the RTCA software. Cell Index was used to measure the change in the electrical impedance divided by background value, which represents cell status. Each assay was biologically performed in triplicate.

### Conditioned media

Cells were cultured in medium without serum for 24 h, and then media were collected and briefly centrifuged. The resulting supernatants were used either immediately or were frozen at -80°C until needed.

### Immunohistochemistry staining on FFPE tissues

Immunohistochemistry on formalin-fixed paraffin-embedded tissues was performed using anti-DNMT1 antibody from Abcam (Cambridge, MA) overnight at a dilution of 1:500 and were stained using automated staining platform (Ventana). Envision + polymer (ready to use; Dako) was used as a secondary antibody. Color was developed with 3,3′-diaminobenzidine (DAB) and instant hematoxylin (Shandon) was used for counterstaining.

### Orthotopic tumor xenografts

Animal experiments were approved by the KFSH&RC institutional Animal Care and Use Committee (ACUC) and were conducted according to relevant national and international guidelines. 10 female nude mice were randomized into 2 groups and breast cancer orthotopic xenografts were created by coimplantation of the MDA-MB-231 cells (2 × 10^6^) with TCF64-orf (TCTorf) or TCF64-c (TCTC) cells (4 × 10^6^) under the nipple of each mouse. Tumour size was measured with a caliper using the following formula (Length X Width X Height).

### Statistical analysis

Statistical analysis was performed by student's *t*-test and *P* values of 0.05 and less were considered as statistically significant.

## SUPPLEMENTARY MATERIALS FIGURES


