# Supplementary material for: The DNA methyl-transferase protein DNMT1 enhances tumor-promoting properties of breast stromal fibroblasts
Source: Oncotarget. 2017 Dec 18;9(2):2329–43. doi: 10.18632/oncotarget.23411 (PMC5788643; doi:10.18632/oncotarget.23411)
Supplement: Supplementary file 1 [file oncotarget-09-2329-s001.pdf]

The DNA methyl-transferase protein DNMT1 enhances tumor-promoting properties of breast stromal fibroblasts

SUPPLEMENTARY MATERIALS

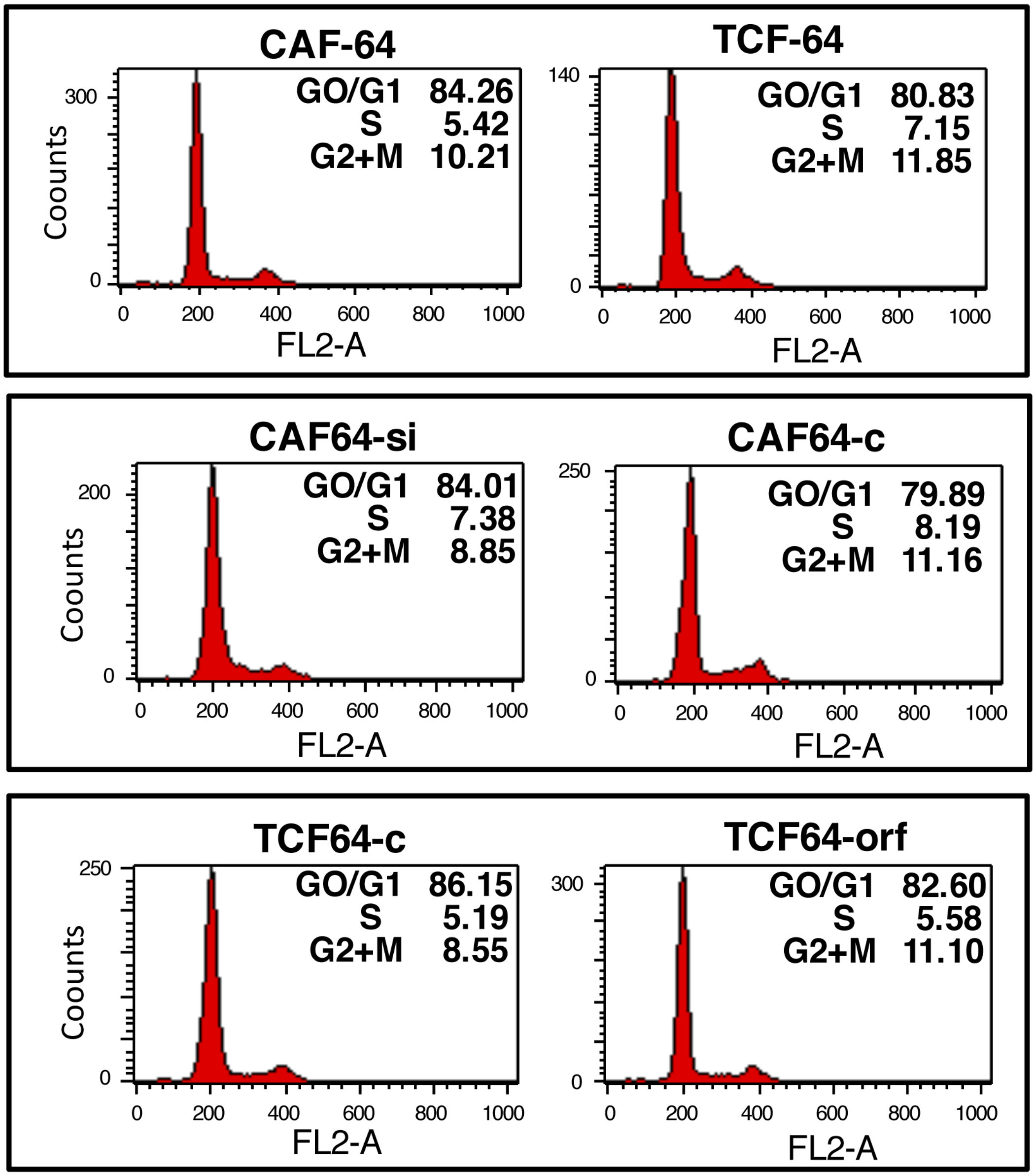

Supplementary Figure 1: Cell cycle distribution of DNMT1-proficient and -deficient breast fibroblasts. Exponentially growing cells were fixed and stained with propidium iodide, and then cell cycle was analyzed by flow cytometry. The numbers indicate the proportion of cells in the indicated phases of the cell cycle.

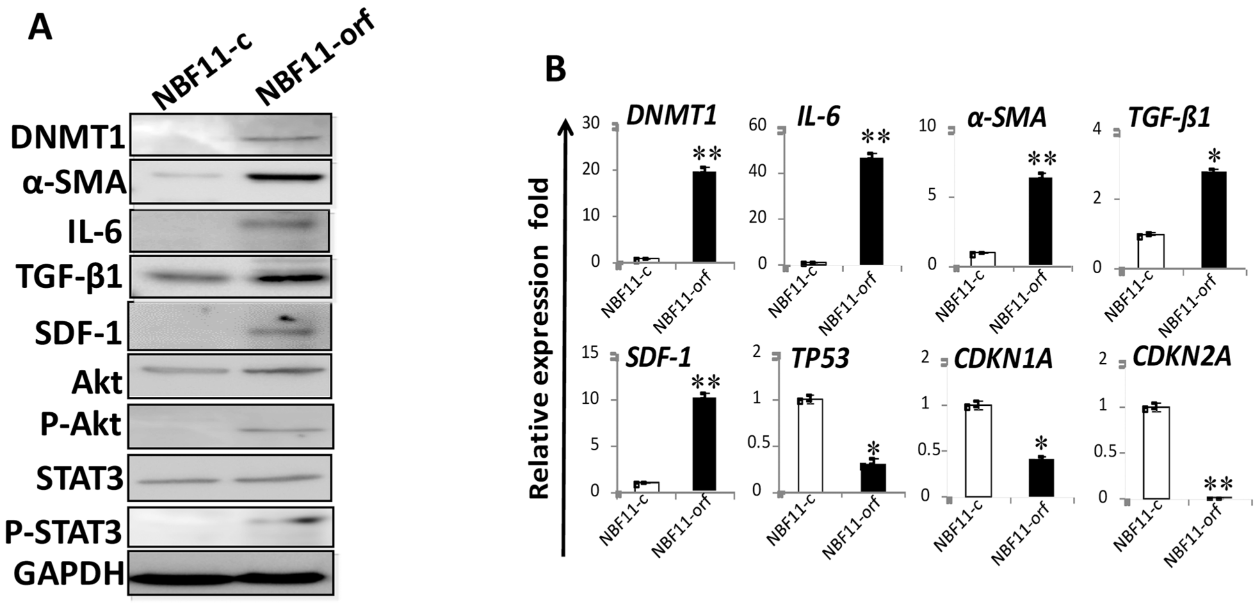

Supplementary Figure 2: Ectopic expression of *DNMT1* activates breast stromal fibroblasts. NBF-11 cells were transfected with a plasmid bearing the DNMT1-ORF (NBFll-orf) or an empty vector (NBFll-c). **(A)** Whole cell lysates were prepared from the indicated cells, and were used for immunoblotting analysis using antibodies against the indicated proteins. **(B)** Figure legends are as in Figure 3C. Error bars represent mean±S.D (n=3). *, P<0.05; **, P<0.003.

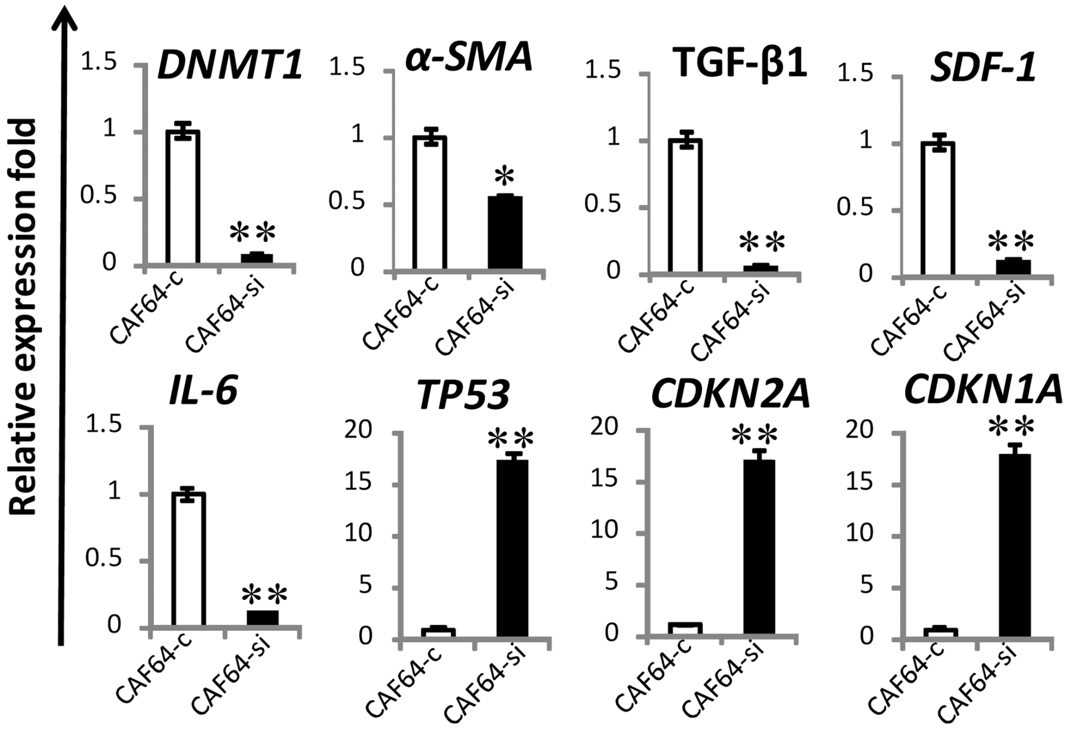

Supplementary Figure 3: DNMT1 down-regulation suppresses myofibroblasts. Total RNA was extracted from the indicated cells and the mRNA levels of the indicated genes were assessed using qRT-PCR. Error bars represent mean±S.D (n=3). *, P=0.007; **, P<0.001.

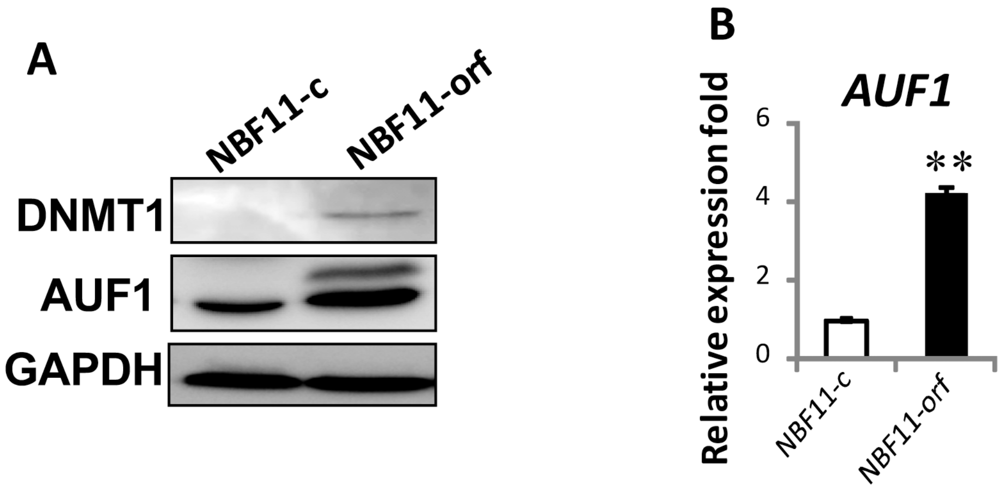

Supplementary Figure 4: DNMT1 positively controls AUF1 expression. NBF-11 cells were transfected with a plasmid bearing the *DNMT1* ORF (NBF11-orf) or an empty vector (NBF11-c). **(A)** Whole-cell lysates were prepared from the indicated cells, and were used for immunoblotting analysis. **(B)** Total RNA was extracted and the AUF1 mRNA level was assessed using qRT-PCR. Error bars represent mean±S.D (n=3). **, *P*=0.01.
